# Gross Nitrogen Mineralization in Surface Sediments of the Yangtze Estuary

**DOI:** 10.1371/journal.pone.0151930

**Published:** 2016-03-18

**Authors:** Xianbiao Lin, Lijun Hou, Min Liu, Xiaofei Li, Guoyu Yin, Yanling Zheng, Fengyu Deng

**Affiliations:** 1 College of Geographical Sciences, East China Normal University, Shanghai 200241, China; 2 State Key Laboratory of Estuarine and Coastal Research, East China Normal University, Shanghai 200062, China; Auckland University of Technology, NEW ZEALAND

## Abstract

Nitrogen mineralization is a key biogeochemical process transforming organic nitrogen to inorganic nitrogen in estuarine and coastal sediments. Although sedimentary nitrogen mineralization is an important internal driver for aquatic eutrophication, few studies have investigated sedimentary nitrogen mineralization in these environments. Sediment-slurry incubation experiments combined with ^15^N isotope dilution technique were conducted to quantify the potential rates of nitrogen mineralization in surface sediments of the Yangtze Estuary. The gross nitrogen mineralization (GNM) rates ranged from 0.02 to 5.13 mg N kg^-1^ d^-1^ in surface sediments of the study area. The GNM rates were generally higher in summer than in winter, and the relative high rates were detected mainly at sites near the north branch and frontal edge of this estuary. The spatial and temporal distributions of GNM rates were observed to depend largely on temperature, salinity, sedimentary organic carbon and nitrogen contents, and extracellular enzyme (urease and L-glutaminase) activities. The total mineralized nitrogen in the sediments of the Yangtze Estuary was estimated to be about 6.17 × 10^5^ t N yr^-1^, and approximately 37% of it was retained in the estuary. Assuming the retained mineralized nitrogen is totally released from the sediments into the water column, which contributed 12–15% of total dissolved inorganic nitrogen (DIN) sources in this study area. This result indicated that the mineralization process is a significant internal nitrogen source for the overlying water of the Yangtze Estuary, and thus may contribute to the estuarine and coastal eutrophication.

## Introduction

Estuarine and coastal ecosystems are important transitional zones between terrestrial and ocean ecosystems, playing a significant role in nitrogen biogeochemical cycle [1]. In recent decades, increasing nitrogen loadings driven by human activities (e.g., nitrogen fertilizer production and fossil fuel combustion) have greatly changed the balance of the nitrogen cycle in estuarine and coastal environments [2], and thus caused numerous environmental issues, such as widespread eutrophication, hypoxia and anoxia, loss of biodiversity, and increased harmful algal blooms [3–5]. Sediments are important sites for organic matter accumulation and nutrient cycling in estuarine and coastal environments [6]. Nitrogen mineralization is a critical biogeochemical process that transfers from organic nitrogen to inorganic forms by heterotrophic microorganism and extracellular enzymes [7], for which it serves as both an energy source and as microbial metabolites [8]. The process of sediment nitrogen mineralization is closely associated with sediment nitrogen supplying capacities and nitrogen loss, which has the important ecological meanings to maintain ecosystem health [9]. However, numerous researches have reported that nitrogen mineralization of sediments is a potential source of dissolved inorganic nitrogen (DIN) releasing into overlying water [8, 10–12]. In oxygen-depleted zones with high organic matter, mineralization of organic matter, which is the major source of ammonium (NH_4_^+^-N), results in NH_4_^+^-N accumulation [1, 13]. Therefore, nitrogen mineralization is likely an important nitrogen internal source and contributes to exacerbation of eutrophication in these ecosystems. Quantifying the internal nitrogen mineralized from sedimentary organic matter may have potential implications for understanding the nitrogen budgets in estuarine and coastal ecosystems.

The Yangtze Estuary is located in the industrial and economic center of China. As a crucial biogeochemical filter at the land-ocean interface, this estuary is affected strongly by anthropogenic nutrient inputs. Since the 1980s, the Yangtze Estuary has received substantial anthropogenic nitrogen from agricultural activities, domestic and industrial wastewater discharge within the river basin [14, 15], which leads to severe eutrophication, red tides and seasonal hypoxia [15, 16]. Thus, the biogeochemical cycle of N is of great concern in the Yangtze Estuary [11, 17, 18]. In recent years, the nitrogen transformation processes, such as nitrification, denitrification, anammox and DNRA, have been examined [11, 17, 19, 20], however no reports regarding sedimentary N mineralization are currently available for this area. In this study, sediment-slurry incubation experiments combined with ^15^N isotope dilution technique were conducted to quantify the gross nitrogen mineralization (GNM) rates in the Yangtze Estuary. Environmental factors and extracellular enzymes activities were determined to elucidate their correlations with the GNM rates. We also determined the percentage of NH_4_^+^-N mineralized to reveal the potential contribution of sedimentary organic matter mineralization to the nitrogen budget in the overlying water of the Yangtze Estuary.

## Materials and Methods

### Study area

The Yangtze Estuary is located in the center of China’s coastal zone, which covers an area of about 8500 km^2^. It has a typical subtropical monsoon climate, with a mean annual temperature of about 15°C and a mean annual precipitation of about 1004 mm [21]. This estuary is subject to a semi-diurnal tide, with a tidal range from about 2.5 to 4.6 m [22]. Although the construction of the Three-Gorge Dam and the protection of ecological environments have resulted in a significant decrease in the flux of suspended sediment from the Yangtze River Basin in recent years [23], a substantial amount (approximately 2 × 10^8^ t) of suspended sediment is still transported into the estuary and its adjacent areas by the Yangtze River each year. In addition, approximately 1.2 × 10^7^ t of particulate organic matter associated with suspended sediment is also carried to the estuary, of which a large portion is deposited in the study area [24]. This suggests that the mineralization of sedimentary organic matter would be a potential nutrient source for the water columns of the Yangtze Estuary.

### Sediment sample collection

**Fig 1:**
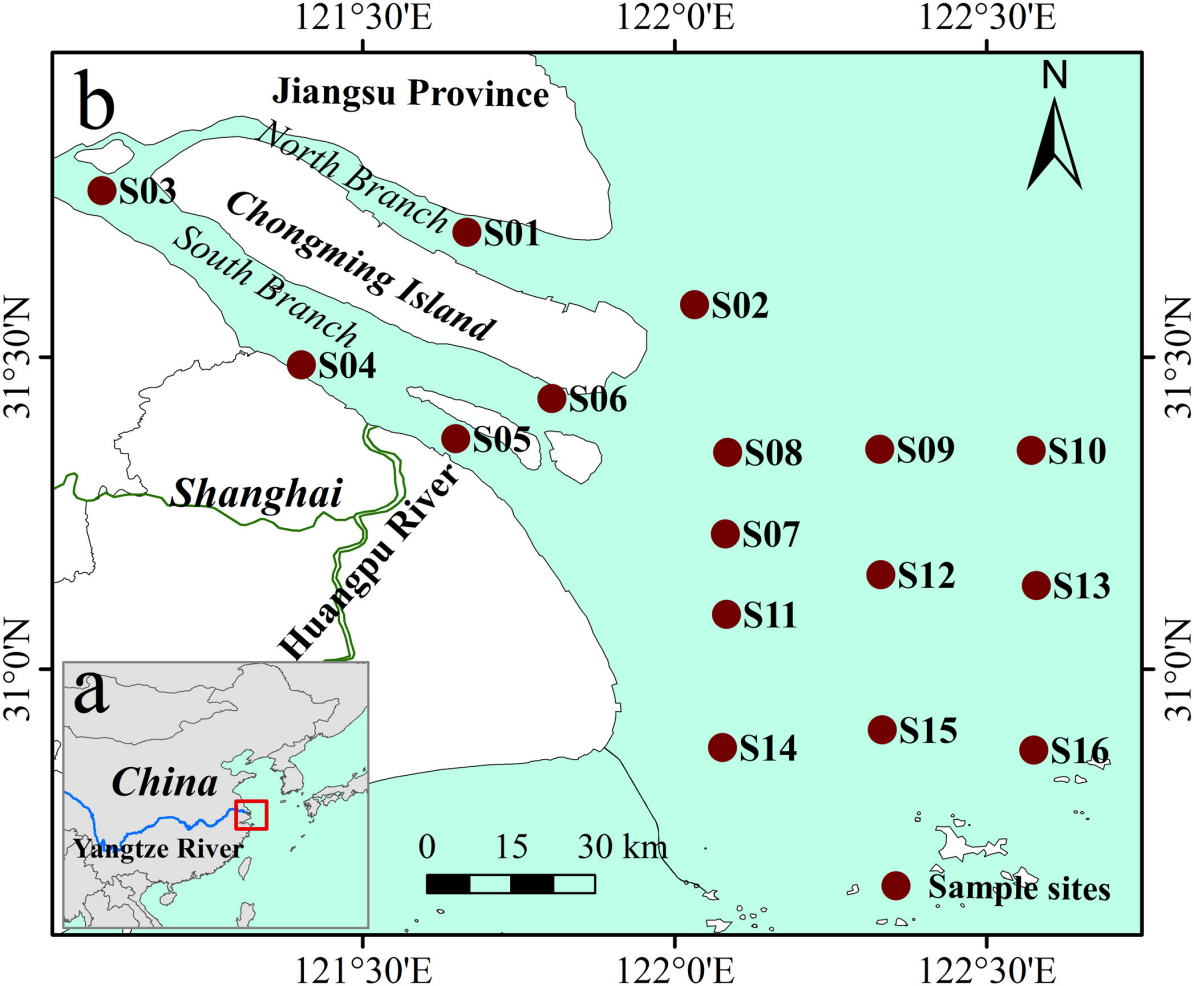
Study area. This figure shows the location of the Yangtze Estuary and the sampling sites.

Field investigations were conducted in July 2013 and January 2014, respectively. Triplicate surface sediments (0–5 cm) were collected from 16 sites (Fig 1) by sub-coring the box corers with PVC tubes. The collected sediment samples were sealed in sterile plastic bags and stored on board at 4°C. After sediment samples were taken to the laboratory, sediment in each core was completely homogenized under helium to produce one composite sample. One portion of the mixed sediment was immediately incubated via slurry experiments to measure GNM rates, while the other portion was examined for sediment physiochemical characteristics. Our studies did not involve endangered or protected species, and collections were only made from public access areas, no specific permits were required to collect sediment samples from these locations/activities.

### Sediment-slurry incubation experiments

In this study, the GNM rates were measured by slurry incubation experiments combined with ^15^N isotope dilution technique [25]. Briefly, slurries were made with sediment samples and site benthic water at a volume ratio of 1:5. The slurries were stirred by magnetic stirrer and purged by helium for approximately 30 minutes to reach an anaerobic condition, and then transferred into 12-mL gastight borosilicate vials (Labco Exetainer) under helium condition. The slurry bottles were subsequently placed on a shaker table (150 rpm) and pre-incubated in dark at near *in situ* temperature for 12 h to achieve stable conditions. After the pre-incubation, the slurry bottles were spiked with ^15^NH_4_^+^-N (final concentration ca. 2 μg ^15^N g^-1^, final % ^15^N ca. 5–10%, depending on the initial NH_4_^+^-N contents) [17]. One half of the replicates were immediately poisoned with 100 μL of saturated HgCl_2_ solution and designated as initial samples. The remaining slurries were shaken (150 rpm) and incubated for approximately 24 h. At the end of incubations, these remaining slurries were also poisoned with HgCl_2_, as described for the initial samples. The initial and final slurry samples were extracted with 2 M KCl solutions for about 1 h, centrifuged at 4,000 rpm for 15 min, and the supernatants were decanted and filtered through 0.22 μm poresize filter for analyses of total NH_4_^+^-N and ^15^NH_4_^+^-N concentrations. Total NH_4_^+^-N concentrations in extractants were determined using continuous-flow nutrient analyzer (SAN Plus, Skalar Analytical B.V., the Netherland). Concentrations of ^15^NH_4_^+^-N in extractants were measured by a new developed Oxidation/MIMS (OX/MIMS) method [26]. In brief, ^15^NH_4_^+^-N was oxidized into dinitrogen gas with hypobromite iodine solution, and the oxidized products (^29^N_2_ and ^30^N_2_) were determined with membrane inlet mass spectrometer (MIMS).

The rates of GNM were calculated using the following equation [27]:

$$m=\frac{M_{0}-M_{1}}{t}\times \frac{\text{log}(H_{0}M_{1}/H_{1}M_{0})}{\text{log}(M_{0}/M_{1})}$$ (1)

where *m* (mg N kg^-1^ dry sediment d^-1^) is the rates of GNM; *t* (d) is the incubation time; *M*_*0*_ and *M*_*1*_ (mg N kg^-1^ dry sediment) are the total NH_4_^+^-N concentrations in the initial and final slurry samples, respectively; *H*_*0*_ and *H*_*1*_ (mg N kg^-1^ dry sediment) are the ^15^NH_4_^+^-N concentrations in the initial and final slurry samples, respectively. In addition, the percentage of NH_4_^+^-N mineralized per day was defined as the GNM rates divided by sediment nitrogen contents and multiplied by 100.

### Sediment enzyme assay

Urease, L-glutaminase and L-asparaginase are important amidohydrolase responsible for nitrogen mineralization, which have been generally used to predict nitrogen mineralization in soils [28, 29]. The activities of these enzymes were determined using the methods from Frankenberger and Tabatabai [30], Muruganandam et al. [29] and Segnini de B et al. [31] with slight modifications. Briefly, they were assayed using 5 g of freeze-dried sediment with their respective substrate, and incubated for 24 h at 37°C. After incubation, developing agents were added into the extracted enzyme solutions for colorimetric analyses. Absorbance in these assays was measured using a colorimetric plate reader (SpectraMax M5 Microplate Spectrophotometer; Molecular Devices Corporation, Sunnyvale, CA). Each assay contained substrate blank and sample blank receiving substrate and deionized water, respectively. The linear regressions between absorbance and standard concentrations were used to calculate the activities of these enzymes. However, the measured L-asparaginase activities in all sediment samples were close to the substrate and sample blank values, so only urease and L-glutaminase activities are presented in this study.

### Determination of environmental parameters

After sampling, sediment temperature and bottom water salinity were measured on board with portable electronic thermometer and YSI Model 30 salinity meter, respectively. Sediment grain size was measured using LS 13320 Laser grain sizer analyzer. Sediment pH was determined with Mettler-Toledo pH meter, after sediment was mixed with CO_2_-free deionized water at a volume ratio of 1:2.5 [20]. Sediment water content was quantified from the amount of weight lost from a known amount of wet sediment that had been dried at 80°C to a constant value. Contents of exchangeable NH_4_^+^-N, nitrate (NO_3_^−^N), and nitrite (NO_2_^−^N) in sediments were extracted with 2 M KCl and determined with continuous-flow nutrient analyzer [18]. Contents of total sediment organic carbon (TOC) and nitrogen (TN) were measured with elementary analyzer (VarioELIII) after removing carbonate by leaching with 0.1 M HCl [32]. These data on sediment characteristics are provided in Supporting Information (S1 Table).

### Statistical analysis

In this study, all statistical analyses were performed with the software SPSS19.0. The relationships of GNM rates with environmental variables and extracellular enzyme activities were determined by Pearson’s correlation and partial correlation analyses. One-way analysis of variance (ANOVA), followed by Tukey’s HSD test, was performed to examine whether temporal and spatial changes in obtained data were statistically significant, and all of them met the assumptions for parametric tests.

## Results

### Spatial and temporal variations of GNM rates

**Fig 2:**
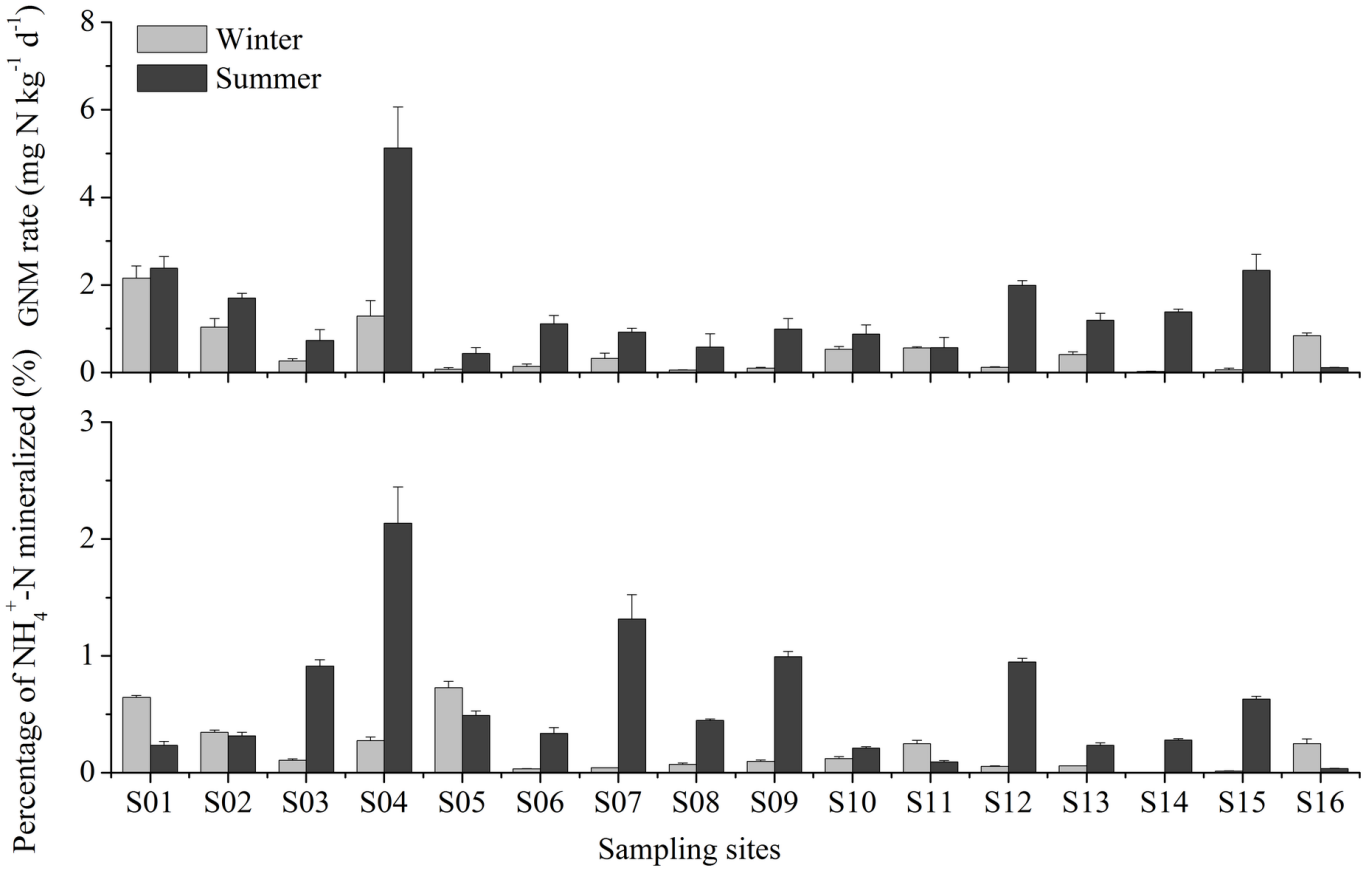
GNM rates and percentages of NH4+-N mineralized per day in surface sediments of the Yangtze Estuary. Vertical bars denote standard error of triplicate samples.

In the study area, the GNM rates of sediments ranged from 0.02 to 5.13 mg N kg^-1^ d^-1^ (Fig 2). A significant seasonal variation in the GNM rates was observed (one-way ANOVA, *P* = 0.007). In general, the GNM rates were higher in summer (0.44–5.13 mg N kg^-1^ d^-1^) than in winter (0.02–2.26 mg N kg^-1^ d^-1^). Meanwhile, there was a remarkable spatial difference in the GNM rates among the sampling sites (one-way ANOVA, *P* < 0.0001). In summer, the lowest GNM rate was detected at site S05, while the highest GNM rate was observed at S04. In winter, the highest GNM rate appeared at site S01, whereas the lowest GNM rate occurred at site S14. Overall, irrespective of season relatively high rates of GNM were detected mainly at sites near the north branch and frontal edge of this estuary.

The estimated percentage of NH_4_^+^-N mineralized per day varied between 0.01% and 2.14% in the study area. This percentage showed a remarkable seasonal variation (one-way ANOVA, *P* = 0.008). A relatively higher percentage was observed in summer (0.03–2.14%) than in winter (0.01–0.76%) (Fig 2). In addition, a significant spatial difference in the percentage of NH_4_^+^-N mineralized per day was observed among the sampling sites (one-way ANOVA, *P* = 0.004). In summer, the highest percentage occurred at site S04, while the lowest percentage appeared at site S11. In winter, the highest and lowest percentages were detected at sites S14 and S05, respectively.

### Activities of extracellular enzymes

**Fig 3:**
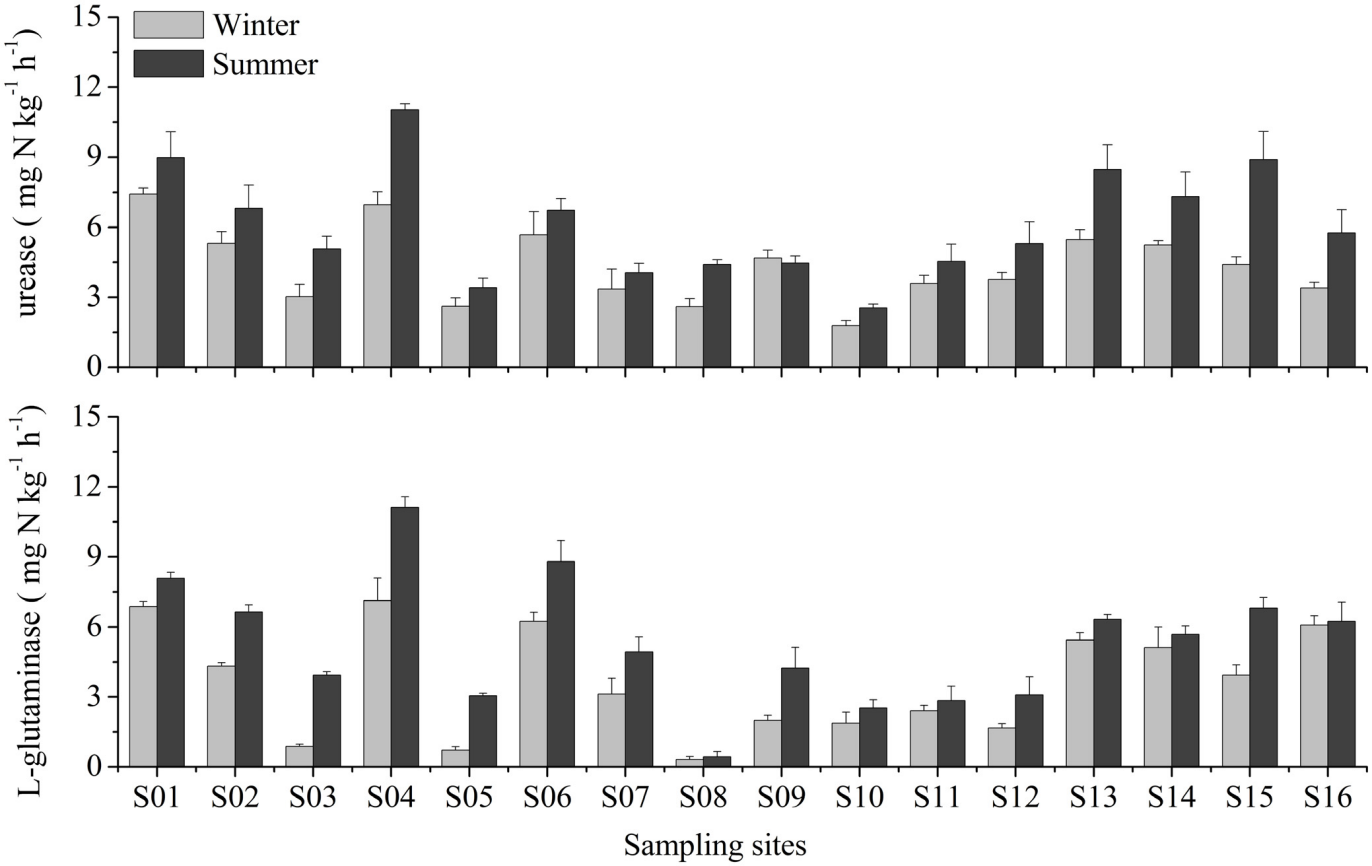
Activities of urease and L-glutaminase in surface sediments of the Yangtze Estuary. Vertical bars denote standard error of triplicate samples.

**Fig 4:**
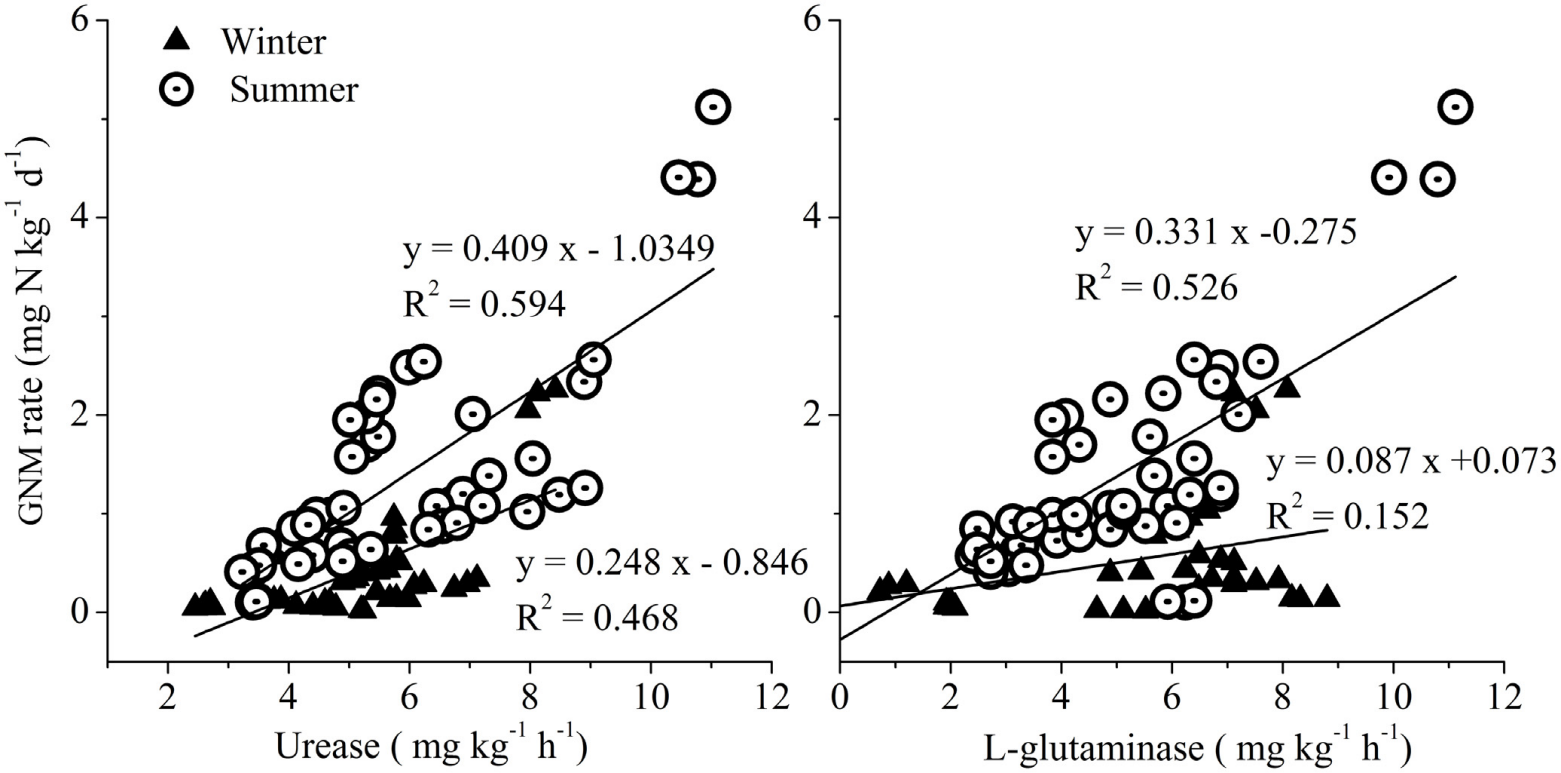
Relationships between the extracellular enzymes activities (urease and L-glutaminase) and GNM rates in surface sediments of the Yangtze Estuary.

The activities of urease ranged from 2.55 to 11.03 mg kg^-1^ h^-1^ and from 1.79 to 7.42 mg kg^-1^ h^-1^ in summer and winter, respectively (Fig 3). One-way ANOVA analyses showed that there were remarkable spatial and seasonal differences in the urease activities in the study area (*P* < 0.01). The activities of L-glutaminase varied from 0.43 to 11.12 mg kg^-1^ h^-1^ in summer and from 0.32 to 7.12 mg kg^-1^ h^-1^ in winter (Fig 3). A significant spatial difference in the L-glutaminase activities was observed among the sampling sites (one-way ANOVA, *P* < 0.0001). However, no seasonal variation was detected for the L-glutaminase activities (one-way ANOVA, *P* > 0.05). Pearson’s correlation analyses indicated that the GNM rates were observed to relate closely with the activities of both urease and L-glutaminase (*P* < 0.01 in all correlations, Fig 4).

### Environmental factors affecting GNM rates

Pearson correlation analyses indicated that the summer GNM rates were significantly related with all environment factors except for sediment temperature, while the winter GNM rates were correlated negatively with the sand, salinity and NH_4_^+^-N but positively with sediment water content, TN, TOC and silt (Table 1). Correlations of environmental factors with GNM rates were also examined through partial (controlling for TOC or TN) correlation analysis. In summer, the GNM rates were only significantly correlated with salinity (*r* = 0.34, *P* < 0.05) after controlling for covariates with TOC via partial correlation, and there were significant relationships of the GNM rates with TOC (*r* = 0.30, *P* < 0.05), salinity (*r* = 0.37, *P* < 0.01), sand (*r* = 0.38, *P* < 0.01) and clay (*r* = -0.30, *P* < 0.05) after controlling for TN. In winter, the GNM rates after controlling for TOC were positively correlated with TN (*r* = 0.46, *P* < 0.01) and salinity (*r* = 0.32, *P* < 0.05), but unrelated to other factors (*P* > 0.05). However, the winter GNM rates were not significantly related with any factors (*P* > 0.05) (except for salinity) after controlling for TN.

**Table 1:** Pearson’s Correlation and Partial Correlation Analyses (Controlling for TOC or TN) between GNM Rates and Environmental Factors.

Parameters	GNM Rates (Summer)			GNM Rates (Winter)		
	Pearson	Partial (TOC)	Partial (TN)	Pearson	Partial (TOC)	Partial (TN)
**Temperature**	0.21	0.13	0.14	0.21	0.16	0.19
**Water content**	0.49**	0.24	0.16	0.40**	0.21	0.13
**Salinity**	-0.53**	-0.34*	-0.37**	-0.47*	-0.32*	-0.29*
**NH**_**4**_^**+**^**-N**	0.30*	0.23	0.19	-0.41**	-0.28	-0.21
**NO**_**3**_^**-**-^**N**	-0.41**	-0.26	-0.24	-0.28	-0.08	-0.20
**TN**	0.46**	0.13	--	0.72**	0.46**	--
**TOC**	0.50**	--	0.30*	0.62**	--	0.04
**Clay**	0.42**	0.21	0.38**	0.27	0.01	-0.07
**Silt**	0.34*	0.04	0.21	0.38**	0.16	0.14
**Sand**	-0.40**	-0.12	-0.30*	-0.35*	-0.11	-0.08

* Significant at *P* < 0.05.

** Significant at *P* < 0.01.

## Discussion

In this study, the spatial and seasonal variations of nitrogen mineralization were examined in surface sediments of the Yangtze Estuary, which provides new insights into microbial nitrogen transformations in the estuarine and coastal environments. Many studies have shown that sediment nitrogen mineralization is associated closely with environmental factors [7, 9, 10, 29]. Temperature is generally considered an important factor responsible for nitrogen mineralization, because it can directly affect microbial metabolism [33]. For instance, it has been demonstrated that GNM rates were significantly higher at 40°C than at 25 and 5°C, with a Q_10_ (fractional change in rate with a 10°C increase in temperature) of 6.5–11 [34]. In the present work, the GNM rates were also observed to be higher in the warm (July) than in the cool (January) season, which is likely attributed to the seasonal temperature change [35]. Meanwhile, it was observed that the percentages of NH_4_^+^-N mineralized per day were also significantly higher in summer than in winter (one-way ANOVA, *P* = 0.008). This observation further implied the importance of temperature in regulating the seasonal variations of GNM rates in this study area. Salinity is considered as a stressor for physiological activity of microorganisms [36]. Previous studies have shown that salinity has a negative influence on nitrogen mineralization [35, 37, 38]. In this study, we also observed a significantly negative correlation between the GNM rates and salinity after controlling for TOC or TN both in summer and winter (Table 1). The decrease of nitrogen mineralization with increasing salinity was likely because high salinity may cause a physiological stress on bacteria involved in the nitrogen mineralization [39]. Our sediments were collected in an interacting zone of both fresh and saline water environments, with a salinity ranging from 0.14 to 33‰. Therefore, the fluctuation of salinity is an important factor influencing sediment nitrogen mineralization in this study area.

Hydrodynamic conditions can regulate re-suspension and remobilization of surface sediments and thus influence the quantity and type of buried organic matter in different depositional settings of estuarine and coastal ecosystems [40]. The hydrodynamic conditions are governed by the Yangtze River Diluted Water and two southward currents (Yellow Sea Coastal Current and Zhe-Min Coastal Current) [41]. Two southward currents are most active during winter, carrying water and sediments from the Yangtze River southward along the inner shelf. In addition, the Taiwan Warm Current intensifies during summer, and thus significantly weakens the southward transport of sediments along the Zhe-Min coast in summer [42]. Therefore, the fine-grained sediments discharged by the Yangtze River is believed to be deposited first in the estuarine region in summer and then re-suspended and remobilized southward in winter along the Zhe-Min coast. In the study, we observed that surface sediments were generally dominated by silt and clay in summer and by sand in winter (S1 Table). This difference implied a seasonal shift in sediment deposition, resuspention and remobilization of sediments in the estuarine region under the intense hydrodynamic conditions [41, 42]. In addition, we found the significant relationships of sediment grain-size (median size) with sedimentary TOC (*r* = 0.46, *P* < 0.01) and TN contents (*r* = 0.43, *P* < 0.01), extracellular enzymes activities (*r* = 0.25, *P* < 0.05 for urease activities; *r* = 0.21, *P* < 0.05 for L-glutaminase activities) and GNM rates (*r* = 0.37, *P* < 0.01) in the study area. These results showed that the hydrodynamic conditions can alter the physicochemical characteristics of sediments, and further affect the spatiotemporal variations of the GNM rates in the Yangtze Estuary.

Previous studies have reported that L-asparaginase, L-glutaminase and urease are the typical amidohydrolases for soil nitrogen mineralization [28, 29]. In this study, only urease and L-glutaminase activities were detected in all collected sediment samples. Although the measured activities of urease and L-glutaminase were relatively lower than those reported in other ecosystems (11–98 mg N kg^-1^ for urease activities, and 55–142 mg N kg^-1^ for L-glutaminase activities) [29, 43], these activities were strongly correlated with the GNM rates (Fig 4). These relationships indicated that urease and L-glutaminase enzymes may play a significant role in shaping the changes of GNM rates in the estuarine and coastal environments.

Nitrogen mineralization is generally carried out by heterotrophic microorganisms [44]. Thus, the organic carbon is hypothesized to play a key role in regulating the process of nitrogen mineralization, because it serves as an energy source for heterotrophic microbial metabolism [45]. This hypothesis is supported by the significant relationship between the GNM rates and TOC observed in this study. Also, the partial correlation analysis further supported the importance of TOC in controlling the GNM rates in summer, The partial correlation analysis further supported the importance of TOC affecting the GNM rates in summer, it may be likely that the microbial activity of nitrogen mineralization was primarily inhibited by limited energy in summer because microbial metabolism increased with increasing sediment temperature [33, 34]. The nitrogen mineralization depends on TN, but not TOC in winter, this result may be attributed to that the sedimentary TOC was enough for low activity of the microorganisms, and GNM rates strongly depended on the active fraction of organic nitrogen in this season. These were in agreement with several studies, which have demonstrated that nitrogen application significantly increases the microbial biomass and activities as well as accelerates nitrogen mineralization rate [46, 47]. Hence, spatial and temporal variations of GNM rates in the study area also depended on the sedimentary TOC and TN contents.

**Fig 5:**
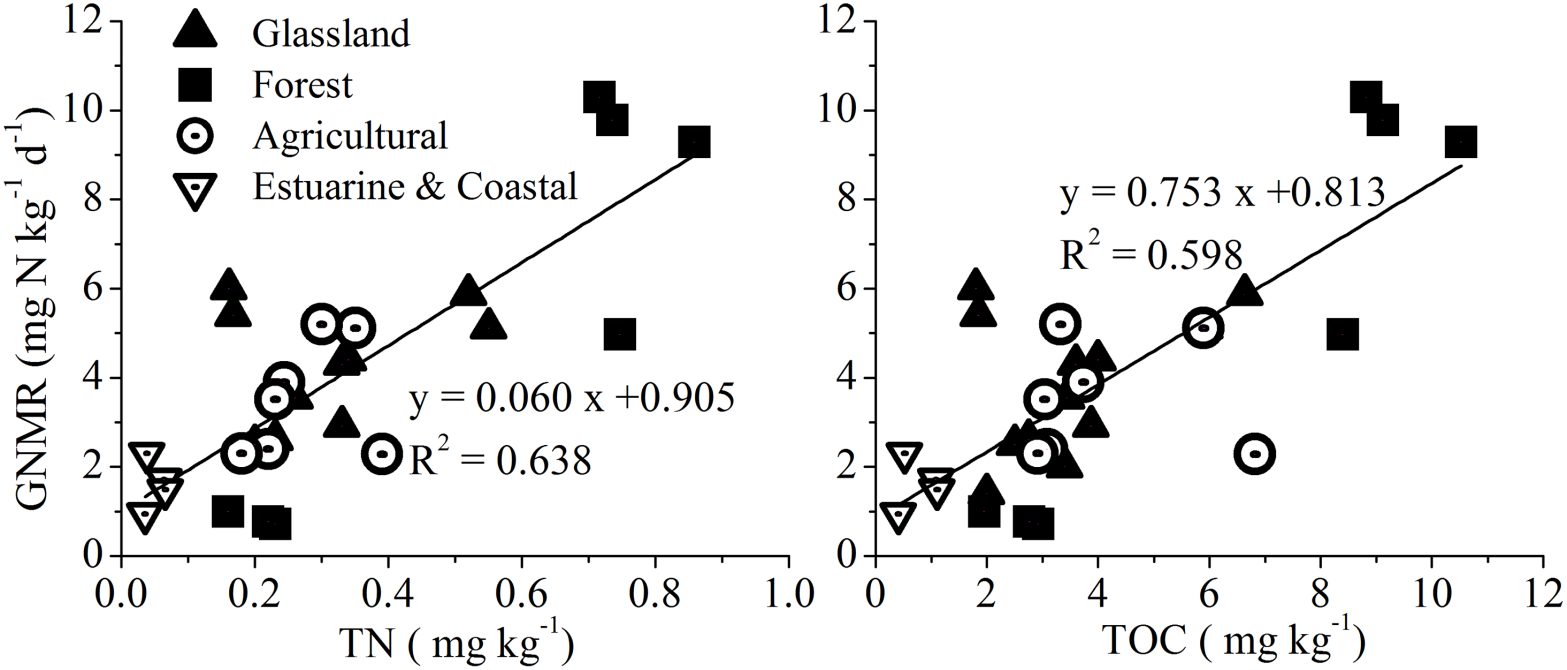
Relationships of GNM rates with soil TOC and TN concentrations in different ecosystems.

To obtain a comprehensive understanding of the GNM rates in the sediments of the Yangtze Estuary, the GNM rates measured in this study are compared with other ecosystems (Table 2). The estimated GNM rates are generally lower than those reported in other ecosystems, which may be attributed to the low concentrations of TOC and TN in the study area. Interestingly, compared with TOC and TN contents in these ecosystems (S2 Table), the GNM rates were found to increase significantly with TOC (*r* = 0.77, *P* < 0.01) and TN (*r* = 0.80, *P* < 0.01) (Fig 5). This comparison also indicates that TOC and TN are important factors responsible for the nitrogen mineralization in natural environments.

**Table 2:** GNM Rates from the Yangtze Estuary and Other Study Areas.

Locations	Sample Type	GNM Rate (mg N kg^-1^ d^-1^)	Authors &Year (Reference)
Lincoln University, New Zealand	Grassland soil	2.50	Zaman et al. 1999a [48]
Cove mountain Farm, USA	Grassland soil	6.53	Corre et al. 2002 [49]
Torup, Sweden	Forest soil	3.90	Bengtsson et al. 2003 [50]
Oklahoma, USA	Forest soil	2.40	Silva et al. 2005 [51]
Oklahoma, USA	Agricultural soil	1.00	Silva et al. 2005 [51]
Fleming, New Zealand	Grassland soil	5.87	Mishra et al. 2005 [52]
Kairanga, New zealand	Grassland soil	5.12	Mishra et al. 2005 [52]
Karapoti, New Zealand	Grassland soil	4.30	Mishra et al. 2005 [52]
Lismore, New Zealand	Grassland soil	4.40	Mishra et al. 2005 [52]
Templeton, New Zealand	Grassland soil	3.54	Mishra et al. 2005 [52]
Waikoikoi, New Zealand	Grassland soil	2.91	Mishra et al. 2005 [52]
Linaria, Canada	Forest soil	5.11	Cheng et al. 2012 [53]
Linaria, Canada	Grassland soil	2.62	Cheng et al. 2012 [53]
Jiangsu Province, China	Marsh sediment (*Spartina anglica*)	1.71	Jin et al. 2012 [54]
Jiangsu Province, China	Marsh sediment (*Phragmites australis*)	1.48	Jin et al. 2012 [54]
Wanmulin Nature Reserve, China	Forest soil (*Castanopsis fargesii*)	2.30	Zhu et al. 2013 [55]
Wanmulin Nature Reserve, China	Forest soil (*Altingia gralilipes*)	2.29	Zhu et al. 2013 [55]
Wanmulin Nature Reserve, China	Forest soil (*Tsoongiodendron Odorum*)	5.20	Zhu et al. 2013 [55]
Wanmulin Nature Reserve, China	Forest soil (*Cunninghamia Lanceolata*)	3.52	Zhu et al. 2013 [55]
Scott, Canada	Grassland soil	2.00	Bedard-Haughn et al. 2013 [56]
Swift Current, Canada	Grassland soil	1.40	Bedard-Haughn et al. 2013 [56]
Yangtze Estuary, China	Estuarine sediment	1.60	Lin et al. 2015 [This study]

The total mineralized nitrogen in the sediments of the Yangtze Estuary can be estimated using the following equation:

F=a⋅ρ⋅S⋅H⋅m (2)

where *F* (t N yr^-1^) is the total mineralized nitrogen in the sediments (*F*) of the Yangtze Estuary; *a* is the unit conversion factor and equivalent to 3.65 × 10^−6^; *ρ* (g cm^-3^) is the sediment dry density, which is about 2.68 g cm^-3^ for this study area [17, 18]; *S* (m^2^) is the area of this study, which was calculated with ArcGIS10.2 software and equivalent to approximately 8200 km^2^; *H* (cm) is the depth of sample collection; *m* (mg N kg^-1^ d^-1^) is the GNM rates. The total mineralized nitrogen was estimated to be approximately 5.96 × 10^5^ t N yr^-1^. However, it should be noted that these GNM rates all derive from stimulated microbial communities and thus could be lower under in situ conditions. Therefore, our calculated rates may not reflect the GNM rates that take place in the field, but rather represent the potential activity in sediments. In order to further assess the net mineralized nitrogen in the surface sediments, the ubiquitous nitrogen loss (including denitrification and anammox) and microbial nitrogen assimilation (including NH_4_^+^-N and NO_3_^−^N assimilation) in the sediments were taken into account [57]. The nitrogen loss was estimated to be approximately 1.92 × 10^5^ t N yr^-1^ for denitrification (*f1*) and 2.30 × 10^4^ t N yr^-1^ for anammox (*f2*) [17, 58]. For microbial nitrogen assimilation (*f3*), it was roughly estimated according to previous studies which reported that nitrogen uptake by microbes removed a fraction of DIN available equivalent to 27% [59, 60]. Here, the microbial nitrogen assimilation was approximately 1.61 × 10^5^ t N yr^-1^. Therefore, the net mineralized nitrogen (*F4*) can be estimated using the following equation:

F4=F−(f1+f2+f3) (3)

**Fig 6:**
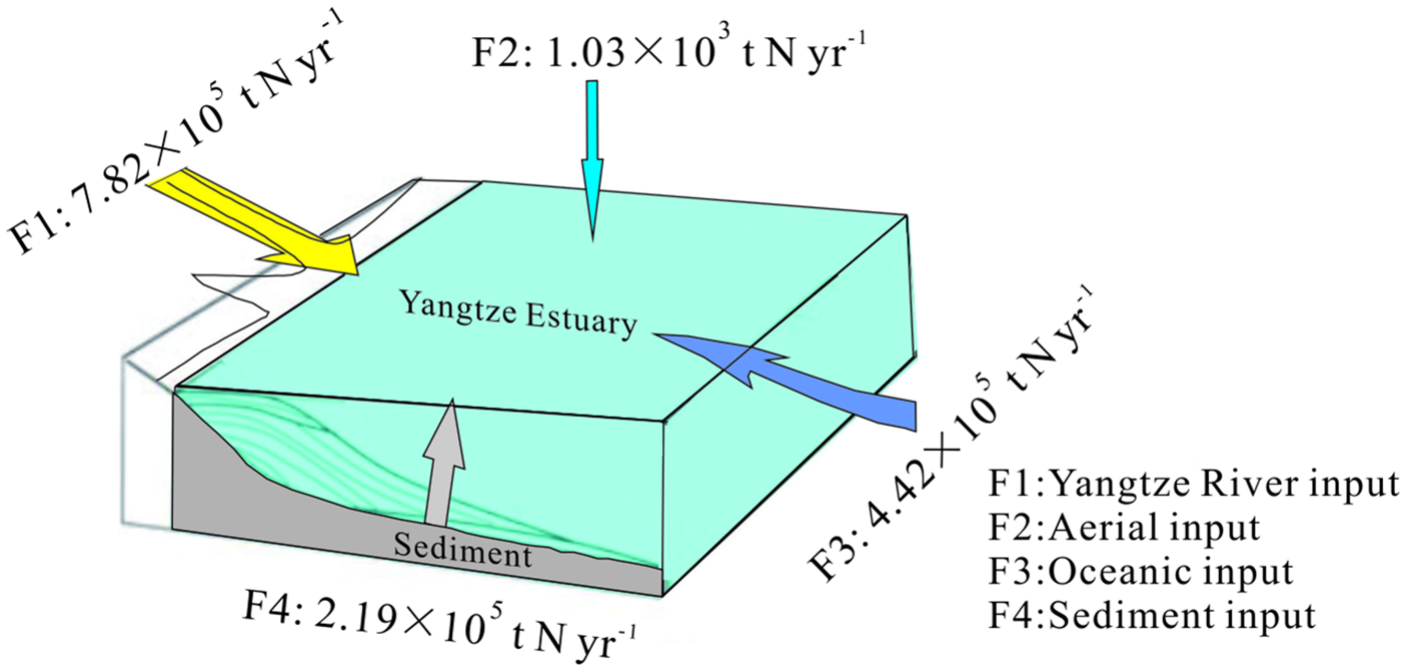
A schematic illustration of different DIN fluxes in the Yangtze Estuary. Data on *F1*, *F2* and *F3* are transformed via changing area according to from Huang et al. (2006), Li (2010), Li et al. (2011), and Kim et al. (2011) [16, 58, 68, 69].

Assuming that the net mineralized nitrogen was totally released from the sediments into the water columns, it was approximately 2.19 × 10^5^ t N yr^-1^, which accounted for 37% of the total mineralized nitrogen. This efflux was within the range reported for other estuarine, coastal, and adjacent offshore environments (S3 Table) [61–68]. Additionally, compared with other DIN sources in the Yangtze Estuary (Fig 6; S4 Table) [58, 69–71], the net mineralized nitrogen is lower than the riverine flux (*F1*, 7.82 × 10^5^–1.21 × 10^6^ t N yr^-1^) and oceanic input (*F3*, ~4.42 × 10^5^ t N yr^-1^), but much higher than the aerial input flux (*F2*, ~1.04 × 10^3^ t N yr^-1^), which contributed 12–15% of total DIN sources (sum of *F1*, *F2*, *F3*, and *F4*) in this study area. This result indicated that nitrogen mineralization is an important internal source of nitrogen in the Yangtze Estuary, which may contribute to estuarine eutrophication and harmful algal blooms.

## Conclusions

The measured GNM rates in surface sediments were greater in summer than in winter, and the higher values were appeared in the north branch and frontal edge of Yangtze Estuary. Meanwhile, GNM rates were related closely to the changes of temperature and salinity, contents of sedimentary organic matter, and activities of extracellular enzymes (urease and L-glutaminase). Additionally, the DIN flux estimated from organic nitrogen of the sediments was approximately 5.96 × 10^5^ t N yr^-1^, and approximately 37% of it was retained in the estuary. Assuming the retained mineralized nitrogen is totally released from the sediments into the water column, which contributed 12–15% of total DIN sources and may accelerate eutrophication in estuarine and coastal ecosystems.

## Supporting Information

S1 Table Physicochemical characteristics of the sediment samples in the Yangtze River Estuary. Values are means (n = 3).
(PDF)

S2 Table Contents of TOC and TN in the Yangtze Estuary and other study areas. NA, no data available.
(PDF)

S3 Table Exchange of dissolved inorganic nitrogen (DIN) across the sediment–water interface in the Yangtze Estuary and other studies. (PDF)

S4 Table Dissolved inorganic nitrogen (DIN) flux in the Yangtze Estuary (this study) and other studies. NA, no data available.
(PDF)
