# Supplementary material for: Gross Nitrogen Mineralization in Surface Sediments of the Yangtze Estuary
Source: PLoS One. 2016 Mar 18;11(3):e0151930. doi: 10.1371/journal.pone.0151930 (PMC4798355; doi:10.1371/journal.pone.0151930)
Supplement: S1 Table — Values are means (n = 3). (PDF) [file pone.0151930.s001.pdf]

S1 Table. Physicochemical characteristics of the sediment samples in the Yangtze River Estuary. Values are means (n = 3).

Sites	Seasons	S01	S02	S03	S04	S05	S06	S07	S08	S09	S10	S11	S12	S13	S14	S15	S16
**Temperature (℃)**	July	29.63	28.94	29.74	29.66	29.57	27.23	27.23	28.62	27.42	27.32	26.71	26.82	26.74	25.31	25.41	25.34
	January	10.67	10.98	10.56	10.72	10.77	11.32	11.32	11.33	11.42	11.52	11.78	11.82	11.83	12.02	12.01	12.05
**Salinity (‰)**	July	9.36	24.77	0.19	0.2	0.14	4.00	4.00	7.18	13.6	20.00	25.50	27.00	21.83	30.23	31.00	33.00
	January	28.00	24.83	0.27	0.22	0.83	0.87	7.23	18.83	25.47	31.00	17.83	27.53	29.00	18.30	24.47	28.53
**pH**	July	8.10	7.85	8.05	8.33	8.49	8.10	8.10	8.11	7.97	8.06	7.86	7.80	7.95	8.14	8.21	8.67
	January	8.53	8.5	8.67	8.76	8.48	7.93	8.06	8.85	8.11	8.58	8.27	8.08	8.11	8.12	8.21	8.31
**Water (%)**	July	58.33	46.25	24.12	31.03	27.82	24.14	24.14	28.25	43.24	34.58	26.9	25.36	39.58	34.27	29.51	59.15
	January	35.53	33.76	29.23	31.97	22.86	22.65	22.65	36.72	25.66	37.67	26.55	26.7	37.68	40.59	44.65	33.83
**TOC(mg g^{-1})**	July	8.11	5.59	0.13	4.90	2.24	3.44	3.44	3.38	7.19	4.71	0.15	0.14	4.23	4.05	5.33	4.82
	January	5.66	4.80	4.15	7.34	3.70	2.56	2.56	6.49	4.57	4.71	4.17	2.31	4.97	4.65	6.18	4.44
**TN(mg g^{-1})**	July	1.06	0.54	0.08	0.24	0.09	0.13	0.13	0.07	0.61	0.50	0.10	0.21	0.37	0.42	0.51	0.31
	January	0.35	0.30	0.25	0.47	0.01	0.07	0.07	0.76	0.23	0.52	0.10	0.22	0.44	0.45	0.71	0.34
**NH_4^+-N (μg g^{-1})**	July	2.57	7.25	3.95	3.82	4.76	3.83	3.83	2.94	6.79	7.80	3.25	3.73	8.06	7.76	11.52	5.05
	January	5.31	5.34	6.06	7.09	8.79	7.86	7.86	4.86	7.18	4.43	7.1	7.18	4.74	4.15	3.71	4.97
**NO_3^--N (μg g^{-1})**	July	0.68	0.39	2.29	0.24	0.66	1.18	1.18	1.08	2.91	1.10	1.17	2.03	0.37	0.90	2.54	0.58
	January	0.61	0.58	5.40	0.89	4.67	2.15	2.15	0.53	1.71	4.52	1.33	1.22	0.65	4.87	0.53	0.67
**Clay (%)**	July	37.3	23.7	32.9	26.2	30.1	32.6	33.6	10.6	17.4	22.1	27.3	4.1	27.9	29.5	29.8	26.3
	January	18.4	12.5	14.2	20.9	5.7	12.5	14.6	3.1	4.0	21.1	7.5	6.6	22.6	20.4	23.2	18.3
**Silt (%)**	July	61.2	68.5	64.9	70.7	68.9	67.2	65.2	26.3	50.6	72.4	71.7	17.9	65.6	68.4	69.8	71.3
	January	49.2	38.7	43.3	61.3	17.8	41.6	43.6	10.5	16.0	50.9	24.35	20.0	44.0	43.7	52.3	51.0
**Sand (%)**	July	1.5	7.8	2.2	3.1	1.0	63.1	63.1	1.2	1.0	2.1	32.0	78.0	0.4	5.5	6.5	2.4
	January	32.4	48.8	42.5	17.8	76.5	86.4	86.4	41.8	68.2	35.9	80.0	73.4	24.5	28.0	33.4	30.7
