# Supplementary material for: Gross Nitrogen Mineralization in Surface Sediments of the Yangtze Estuary
Source: PLoS One. 2016 Mar 18;11(3):e0151930. doi: 10.1371/journal.pone.0151930 (PMC4798355; doi:10.1371/journal.pone.0151930)
Supplement: S2 Table — NA, no data available. (PDF) [file pone.0151930.s002.pdf]

S2 Table. Contents of TOC and TN in the Yangtze Estuary and other study areas. NA, no data available.

**Locations**	**Sample Type**	**TN (%)**	**TOC (%)**	**Authors &Year (Reference)**
Lincoln University, New Zealand	Grassland soil	0.20	2.50	Zaman et al. 1999a [48]
Cove mountain Farm, USA	Grassland soil	0.17	1.83	Corre et al. 2002 [49]
Torup, Sweden	Forest soil	0.24	3.74	Bengtsson et al. 2003 [50]
Oklahoma, USA	Forest soil	0.22	3.08	Silva et al. 2005 [51]
Oklahoma, USA	Agricultural soil	0.16	1.95	Silva et al. 2005 [51]
Fleming, New Zealand	Grassland soil	0.52	6.64	Mishra et al. 2005 [52]
Kairanga, New zealand	Grassland soil	0.55	5.92	Mishra et al. 2005 [52]
Karapoti, New Zealand	Grassland soil	0.33	3.60	Mishra et al. 2005 [52]
Lismore, New Zealand	Grassland soil	0.34	4.00	Mishra et al. 2005 [52]
Templeton, New Zealand	Grassland soil	0.26	3.44	Mishra et al. 2005 [52]
Waikoikoi, New Zealand	Grassland soil	0.33	3.87	Mishra et al. 2005 [52]
Linaria, Canada	Forest soil	0.35	5.90	Cheng et al. 2012 [53]
Linaria, Canada	Grassland soil	0.23	2.75	Cheng et al. 2012 [53]
Jiangsu province, China	Coastal sediment (*Spartina anglica*)	0.06	1.08	Jin et al. 2012 [54]
Jiangsu province, China	Coastal sediment (*Phragmites australis*)	0.07	1.11	Jin et al. 2012 [54]
Wanmulin Nature Reserve, China	Forest soil (*Castanopsis fargesii*)	0.18	2.91	Zhu et al. 2013 [55]
Wanmulin Nature Reserve, China	Forest soil (*Altingia gralilipes*)	0.39	6.82	Zhu et al. 2013 [55]
Wanmulin Nature Reserve, China	Forest soil (*Tsoongiodendron Odorum*)	0.30	3.33	Zhu et al. 2013 [55]
Wanmulin Nature Reserve, China	Forest soil (*Cunninghamia Lanceolata*)	0.23	3.04	Zhu et al. 2013 [55]
Scott, Canada	Grassland soil	NA	3.40	Bedard-Haughn et al. 2013 [56]
Swift Current, Canada	Grassland soil	NA	2.00	Bedard-Haughn et al. 2013 [56]
Yangtze Estuary, China	Estuarine sediment	0.04	0.47	Lin et al. 2015 [This study]
