# Supplementary material for: Gross Nitrogen Mineralization in Surface Sediments of the Yangtze Estuary
Source: PLoS One. 2016 Mar 18;11(3):e0151930. doi: 10.1371/journal.pone.0151930 (PMC4798355; doi:10.1371/journal.pone.0151930)
Supplement: S3 Table — (PDF) [file pone.0151930.s003.pdf]

S3 Table. Exchange of dissolved inorganic nitrogen (DIN) across the sediment–water interface in the Yangtze Estuary and other studies.

Locations	DIN Fluxes (mmol m^{-2} yr^{-1})	Authors & Year (Reference)
Gulf of Lions, France	60~110	Denis et al. (2001) [61]
Swedish West Coast, Swedish	224	Mermillod-Blondin et al. (2005) [62]
Jiaozhou Bay, China	20.41	Liu et al. (2005) [63]
East China Sea, China	146~474	Zhang et al. (2007) [64]
Mandovi Estuary, India	1473	Pratihary et al. (2009) [65]
New Caledonia Lagoon, France	-42~726	Grenz et al. (2010) [66]
Pearl River Estuary, China	-2723~4712	Zhang et al. (2014) [67]
Yangtze Estuary, China	1915	Lin et al. 2015 [This study]
