# Supplementary material for: Gross Nitrogen Mineralization in Surface Sediments of the Yangtze Estuary
Source: PLoS One. 2016 Mar 18;11(3):e0151930. doi: 10.1371/journal.pone.0151930 (PMC4798355; doi:10.1371/journal.pone.0151930)
Supplement: S4 Table — NA, no data available. (PDF) [file pone.0151930.s004.pdf]

S4 Table. Dissolved inorganic nitrogen (DIN) flux in the Yangtze Estuary (this study) and other studies. NA, no data available.

DIN Fluxes Sources (Time)	Temporal Coverage	DIN Fluxes (mol yr^{-1})	Authors &Year (Reference)
Yangtze River input (1959)	NA	1.45×10^{10}	Huang et al (2006) [68]
Yangtze River input (1960s)	NA	1.73×10^{10}	Huang et al (2006) [68]
Yangtze River input (1970s)	NA	1.86×10^{10}	Huang et al (2006) [68]
Yangtze River input (1980s)	NA	5.31×10^{10}	Huang et al (2006) [68]
Yangtze River input (1990s)	NA	1.02×10^{11}	Huang et al (2006) [68]
Yangtze River input (1900-2010)	NA	8.6×10^{10}	Kim et al. (2011) [69]
Yangtze River input (2000s)	Flood and dry season	1.73×10^{11}	Xu et al. (2013) [70]
Yangtze River input (2006)	Four seasons	5.58×10^{10}	Li et al. (2011) [58]
Aerial input (2006)	Four seasons	3.30×10^{10}	Li et al. (2011) [58]
Oceanic input (2006)	Four seasons	1.48×10^{8}	Li et al. (2011) [58]
Sediment input (2014)	Summer and winter	1.56×10^{10}	Lin et al. 2015 [This study]
